# Neural mechanisms of pain processing differ between endurance athletes and nonathletes: A functional connectivity magnetic resonance imaging study

**DOI:** 10.1002/hbm.25659

**Published:** 2021-09-15

**Authors:** Maria Geisler, Alexander Ritter, Marco Herbsleb, Karl‐Jürgen Bär, Thomas Weiss

**Affiliations:** ^1^ Department of Clinical Psychology Friedrich‐Schiller‐University Jena Jena Germany; ^2^ Section of Neurological Rehabilitation, Hans–Berger Department of Neurology Jena University Hospital Jena Germany; ^3^ Department of Sports Medicine and Health Promotion Friedrich‐Schiller‐University Jena Jena Germany; ^4^ Department of Psychosomatic Medicine and Psychotherapy University Hospital Jena Jena Germany

**Keywords:** endurance sport, functional magnetic resonance imaging (fMRI), heat pain, neurosignature of physical pain, pain modulation

## Abstract

Pain perception and the ability to modulate arising pain vary tremendously between individuals. It has been shown that endurance athletes possess higher pain tolerance thresholds and a greater effect of conditioned pain modulation than nonathletes, both indicating a more efficient system of endogenous pain inhibition. The aim of the present study was to focus on the neural mechanisms of pain processing in endurance athletes that have not been investigated yet. Therefore, we analyzed the pain processing of 18 male athletes and 19 healthy male nonathletes using functional magnetic resonance imaging. We found lower pain ratings in endurance athletes compared to nonathletes to physically identical painful stimulation. Furthermore, brain activations of athletes versus nonathletes during painful heat stimulation revealed reduced activation in several brain regions that are typically activated by nociceptive stimulation. This included the thalamus, primary and secondary somatosensory cortex, insula, anterior cingulate cortex, midcingulate cortex, dorsolateral prefrontal cortex, and brain stem (BS). Functional connectivity analyses revealed stronger network during painful heat stimulation in athletes between the analyzed brain regions except for connections with the BS that showed reduced functional connectivity in athletes. Post hoc correlation analyses revealed associations of the subject's fitness level and the brain activation strengths, subject's fitness level and functional connectivity, and brain activation strengths and functional connectivity. Together, our results demonstrate for the first time that endurance athletes do not only differ in behavioral variables compared to nonathletes, but also in the neural processing of pain elicited by noxious heat.

## Graphical abstract

The aim of the present study was to focus on the neural mechanisms of pain processing in endurance athletes that have not been investigated yet. Therefore, we analyzed the pain processing of 18 male athletes and 19 healthy male nonathletes using functional magnetic resonance imaging. Together, our results demonstrate for the first time that endurance athletes do not only differ in behavioral variables compared to nonathletes, but also in the neural processing of pain elicited by noxious heat.

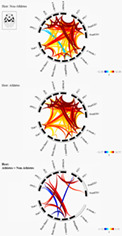

## 1 INTRODUCTION

Pain perception has a profound biological significant in evolution and survival of human being. Thus, pain is an integral part of life and has an important protective function by eliciting unpleasant emotions.

Endurance athletes, however, expose themselves voluntarily to extreme physical exertion often accompanied by acute pain experiences during training or the following days. In contrast to chronic pain patients, who unwillingly suffer from uncontrollable and unpredictable pain, pain perceptions in athletes arise from a completely different context. First of all, it is often voluntarily and self‐elicited by rigorous training. Second, pain experiences do not indicate always‐harmful physical conditions. Moreover, pain experiences accompany every days training schedules for many years. Athletes seem to get adjusted to the necessity to endure painful events. Third, endurance athletes need to learn to cope with the arising pain to get rewarded by new bests or by winning a competition (Flood, Waddington, & Cathcart, 2017). Fourth, the type of sport plays an important role as well. Athletes in contact sports experiencing numerous physical collisions have completely different experiences in comparison to endurance athletes with less acute and unforeseeable pain experiences. Thus, multifarious and sport specific experiences of pain accompany an athlete's career.

Recent studies focusing on endurance athletes showed higher pain tolerance thresholds in this population (Geisler, Herbsleb, Bär, & Weiss, 2020; Geva & Defrin, 2013; Geva, Pruessner, & Defrin, 2017; Tesarz, Schuster, Hartmann, Gerhardt, & Eich, 2012). In a meta‐analysis, Tesarz et al. (2012) revealed that there is a moderate effect for higher pain tolerance thresholds in endurance athletes compared to normally active controls (Hedges' g = 0.65, CI_95%_ [0.42, 0.88]). Examining 19 triathletes and 17 nonathletes, Geva and Defrin (2013) replicated higher pain tolerance thresholds and lower pain ratings in endurance athletes. The question, however, what kind of mechanisms might lead to differences in pain perception in endurance athletes is not answered. Various hypotheses have been proposed such as that repetitive exposure for low intensity pain might induce physical and mental tolerance for pain or that increased baroreflex sensitivity might influence pain tolerances. Some evidence recently indicated that the pain modulation system is more efficient in endurance athletes. In this connection, various authors have shown that endurance athletes have a greater conditioned pain modulation (CPM) effect compared to nonathletes (Flood, Waddington, Thompson, & Cathcart, 2016; Geisler et al., 2020; Geva et al., 2017; Geva & Defrin, 2013). Thus, Freund, Schuetz, Weber, and Birklein (2013) have suggested that endurance athletes develop a more efficient system of endogenous pain inhibition over the years of intensive training. However, the underlying neural mechanisms of pain processing in endurance athletes have not been investigated, yet. One indication of differences in neural pain processing due to chronic effects of exercise was reported in our recent study (Geisler, Eichelkraut, Miltner, & Weiss, 2019). In that study, we analyzed pain processing in 13 endurance athletes in expectation of a run compared to a run‐free control day using functional magnetic resonance imaging (fMRI). Analyzing the data of the run‐free control day in an explorative analysis, we found a negative correlation between the activation of different brain regions that are typically activated by nociceptive stimulation during painful stimulation and the reported training frequency of athletes. This result strengthens the hypothesis that longer endurance training results in longer‐lasting changes in the pain‐modulating system.

The aim of the present study was to investigate neural mechanisms of pain processing in endurance athletes compared to nonathletes. We conducted an fMRI study to physically identical stimuli in athletes versus nonathletes and analyzed the functional connectivity between predefined brain regions that are typically activated by nociceptive stimulation (Wager et al., 2013) during painful heat stimulation using a region of interest (ROI)‐to‐ROI connectivity analysis. We hypothesized H1: lower pain ratings in athletes compared to nonathletes to heat stimuli, but not to warm stimuli. This hypothesis refers to the results of a meta‐analysis (Tesarz et al., 2012) and is therewith based on the most convincing evidence, comparing all three hypotheses. When the H0 (there are no differences in pain ratings between groups) of the H1 can be rejected, we can assume a success of our study manipulation and continue with testing the H2: reduced activation of brain regions that are typically activated by nociceptive stimulation in athletes compared to nonathletes to painful heat stimuli, but not to warm stimuli. This hypothesis has not been tested, yet. As described above, a more efficient system of endogenous pain inhibition is mirrored in a reduction of the activation strength of brain regions that are typically activated by nociceptive stimulation (Geisler et al., 2019). We will focus on bilateral thalamus, bilateral SI and MI, bilateral SII, bilateral anterior and posterior insula, bilateral ACC, bilateral MCC, bilateral PFC and the bilateral brain stem (BS) in this initial study on possible mechanism of altered pain processing in endurance athletes. When the H0 (there are no differences of brain activation to painful heat stimulation between groups) of the H2 can be rejected, we can test our last hypothesis that has neither been tested, yet H3: Different functional connectivity between brain regions that are typically activated by nociceptive stimulation to painful heat stimuli in athletes compared to nonathletes, but not to warm stimuli. On the one hand, we would expect stronger functional connectivity in athletes compared to nonathletes when we assume that athletes have more experiences in pain than nonathletes that might lead to a more often coupled activation of brain regions that are typically activated together by nociceptive stimulation. On the other hand, we would expect lower functional connectivity in athletes between brain regions that influence each other negatively during painful stimulation. When there are differences between athletes and nonathletes in the brain activation strengths or the functional connectivity, we can explore the associations between the subject's fitness level, the brain activation strengths, and the functional connectivity.

## 2 MATERIALS AND METHODS

### 2.1 Participants

Participants were recruited by advertisement posted at the University of Jena, by social networks for runners and triathletes, and by directly contacting run and triathlon clubs in Jena and surroundings. We only included male athletes in the study to reduce variability. Inclusion criteria were as follows: age 18–40 years; body mass index (BMI) 18.5–30 kg/m^2^; no pain disorder; current or past psychiatric or neurological disease; and no contraindication for fMRI scanning. Specific inclusion criteria for athletes were: at least 6 hr/week endurance training for the last 3 years with no sign of exercise dependence risk (total score of the German version of the exercise dependence scale less than 78 (Müller et al., 2013); physical work capacity (PWC) during heart rate (HR) of 150 bpm (PWC150) ≥ 3.0 W/kg. Specific inclusion criteria for nonathletes were: no regular participation in any kind of sports; PWC150 ≤ 2.2 W/kg. The final sample size included 18 male athletes (age: 27.9 ± 4.9 years, BMI: 22.9 ± 1.5 kg/m^2^) and 19 BMI‐ and age‐matched nonathletes (age: 26.1 ± 6.5 years, BMI: 23.8 ± 3.1 kg/m^2^). Detailed comparisons are given in Table 1. Subjects were paid for participation (25 €). Written informed consent was obtained from all participants. The Ethics committee of the Faculty of Social and Behavioral Sciences of the Friedrich Schiller University Jena approved the study (FSV 17/03).

**TABLE 1:** Demographic characteristics of subjects

	Athletes		Nonathletes		*p*
	*M*	*SD*	*M*	*SD*	
*Biographical data*					
Age (years)	27.9	4.9	26.1	6.5	.324
BMI (kg/m^2^)	22.9	1.5	23.8	3.1	.253
Endurance sport (h/week)	10.3	5.7	0	0	**<.001** ^**§**^
Exercise dependence scale	56.6	11.6			
*Aerobic fitness*					
PWC150 (W/kg)	3.5	0.5	1.6	0.3	**<.001**
LT (W/kg)	2.7	0.5	1.1	0.3	**<.001**

*Note*: Group specific mean (*M*) and *standard deviation (SD)* of demographic variables. *p*‐values are given for group comparisons using independent sample *t*‐tests when data were normally distributed and Mann–Whitney *U* test (marked with §) otherwise. The significance was set to *p* < .05 (bold *p*‐values).

Abbreviations: LT, lactate threshold (watt per kg body mass); PWC150, physical work capacity during a heart rate of 150 (watt per kg body mass).

### 2.2 Study design

**FIGURE 1:**
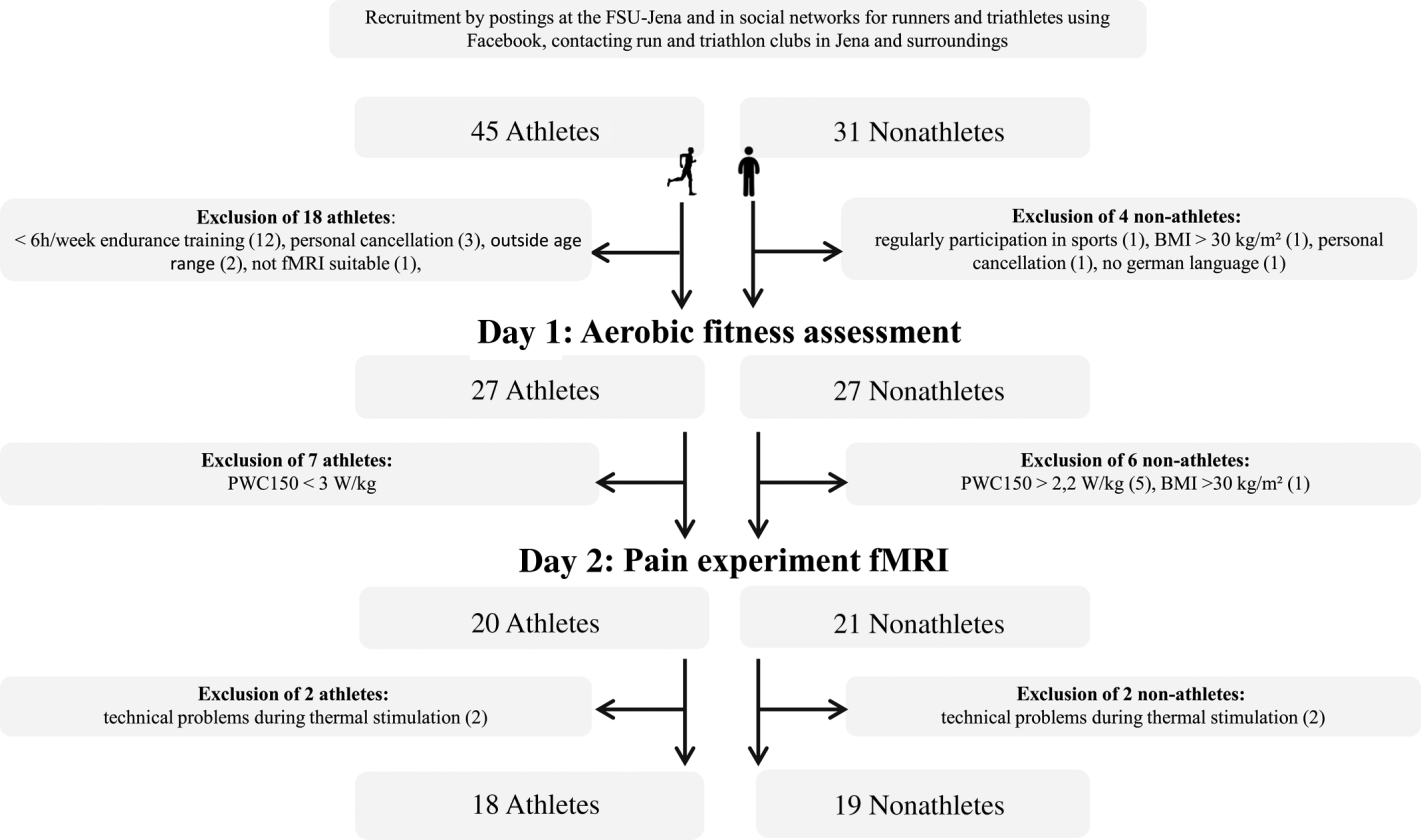
Flow chart of data acquisition. BMI, body mass index; fMRI, functional magnetic resonance imaging; FSU, Friedrich Schiller University; PWC, physical work capacity

**FIGURE 2:**
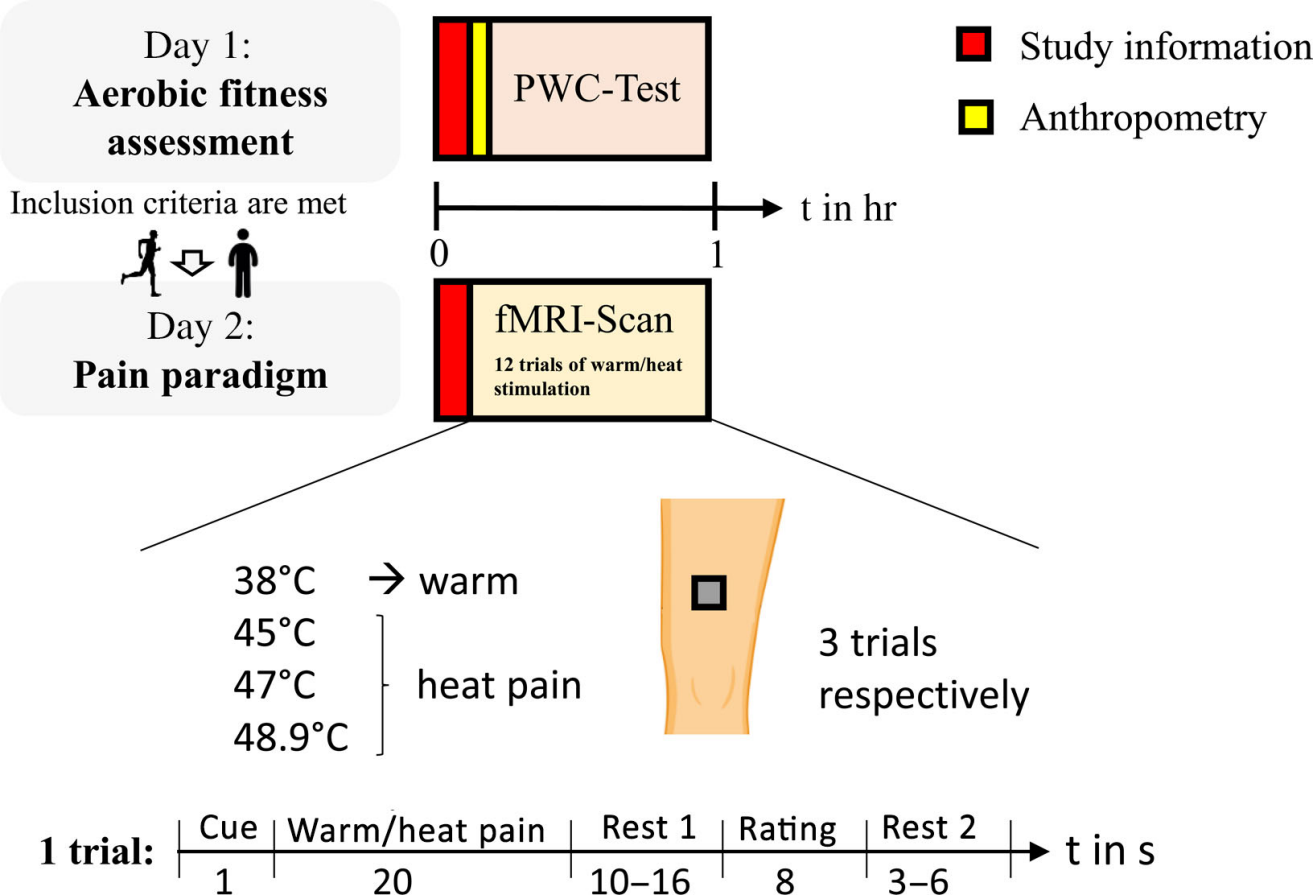
Study design. All participants were investigated at two separate days, that is, Day 1: aerobic fitness assessment, Day 2: Pain paradigm. During the fMRI‐Scan warm/heat pain stimuli were applied. An exemplary trial with durations of the different conditions (numbers below the respective condition) is given. fMRI, functional magnetic resonance imaging; PWC, physical work capacity

All participants were investigated at two separate days, that is, the aerobic fitness was assessed at Day 1, and the pain experiment in the fMRI scanner took place on a second day. The athletes were instructed prior to the study inclusion to maintain their usual training schedule throughout the study period and to avoid a tapering period during this time. The mean time delay between both days was 24 ± 40 days (Figures 1 and 2). Furthermore, all participants filled out several questionnaires at home between study visits assessing attitudes to pain, pain catastrophizing, and other psychological variables that have been shown to modulate pain perception (Beck depression‐inventory‐II [BDI II], Beck, Steer, & Brown, n.d.); (Positive and Negative Affect Schedule [PANAS], Krohne, Egloff, Kohlmann, & Tausch, 1996; Watson, Clark, & Tellegen, 1988); (state and trait anxiety inventory [STAI‐G], Laux, Glanzmann, Schaffner, & Spielberger, 1981); (Life‐Orientation‐Test‐Revised [LOT‐R], Glaesmer, Hoyer, Klotsche, & Herzberg, 2008); (short version of Eysenck Personality Questionnaire‐Revised [EPQ‐RK], Ruch, 1999); (Pain Catastrophizing Scale [PCS], Meyer, Sprott, & Mannion, 2008); (Multidimensional Assessment of Interoceptive Awareness (MAIA], Mehling et al., 2012); (Big‐Five‐Inventory‐10 [BFI‐10], Rammstedt, Kemper, Klein, Beierlein, & Kovaleva, 2013); (Pain Anxiety Symptoms Scale [Pass‐20‐GV], Kreddig, Rusu, Burkhardt, & Hasenbring, 2015). Detailed information on the results of the assessed variables and there associations with pain perception have been reported elsewhere (Geisler et al., 2020).

#### 2.2.1 Aerobic fitness assessment

After subjects had been informed about the study's procedure, anthropometry data were assessed. This included measurements of body height, mass, and skinfold thickness at four sites (biceps, triceps, subscapular, and supra‐iliac) to estimate body fat (Durnin & Womersley, 1974). Subsequently, the aerobic fitness was assessed using a submaximal cycle ergometry test. Exercise testing was performed in the upright position with an electronically braked cycle ergometer (Ergometrics 900, Ergoline, Bitz, Germany). After a resting period of 5 min, where subjects were instructed to sit quietly and relaxed at the cycle ergometer, an incremental bicycle protocol started with the subject pedaling at 25 W (W) for 2 min. The power output was then increased by 25 W every 2 min until the subject reached a target HR of 150 bpm. We encouraged all subjects to maintain a pedaling frequency of 70 rpm throughout the whole test session. HR was continuously recorded using a HR monitor (RS800CX, Polar Electro, Kempele, Finland). The degree of effort exerted by the participants at the end of each workload was determined using the standardized subjective exhaustion 6–20 Borg Scale (Borg, 1982), and capillary blood samples for lactate analyses (Enzymatic‐Amperometric Measuring System, Hitado super GL2 analyzer, Dreihausen, Germany) were taken prior to starting the test as well as at the end of each workload stage.

A special software (ERGONIZER, Freiburg, Germany) was used for the investigator‐independent calculation and was based on an equalizing SPLINE interpolation procedure. The lactate threshold (LT) determined from this interpolated curve over the minimum of the quotient lactate/power output was taken as the start of increase in lactate concentration (Roecker, Schotte, Niess, Horstmann, & Dickhuth, 1998).

To describe aerobic fitness, we determined two submaximal indicators of aerobic capacity:

1. The Physical Working Capacity (PWC‐150), which represents the power output at a HR of 150 beats per minute and was determined using a heart rate‐power output plot.
2. The LT, which represents the first increase in blood lactate concentrations above resting values and demarcates the upper limit of the moderate exercise intensity domain, in which the energy demand is relatively rapidly and almost entirely met by aerobic metabolism.

The determination of these two parameters offers the great advantage compared to parameters like maximum oxygen uptake ($\dot{V}O_{2}$ max) that maximal effort and motivation in subjects are not mandatory and the testing procedure is less risky.

#### 2.2.2 Pain paradigm during fMRI

During fMRI, a pseudo‐random sequence of twelve 20‐s thermal stimuli with different intensities (38, 45, 47, and 48.9°C; three trials each) was applied using a 27 mm diameter fMRI‐compatible Peltier thermode (PATHWAY Model, Contact Heat‐Evoked Potential Stimulator [CHEPS]; Ramat Yishai, Israel). The stimuli were applied on a 4 × 4‐cm square drawn on the subjects' left anterior thigh, dermatome L3. All participants endured the noxious stimulation. The lower end of the square was located medial about 10 cm above the patella. Each trial started with a cue “+.” After presenting the cue for 1 s, the thermal stimulus was administered for 20 s (approximately 1.5‐second ramp up, 17‐s plateau, approximately 1.5‐s ramp down) at the left thigh. After a short delay (10–16 s; Rest 1), participants were asked to rate (8 s) the level of pain of each stimulus on the presented visual analogue scale ranging from 0 = no pain to 100 = unbearable pain. A variable intertrial interval (3–6 s; Rest 2) followed before the start of the next thermal stimulation.

### 2.3 Behavioral data analysis

All data were analyzed using R version 3.4.1 (Team, 2017). Significance levels were set to *p* ≤ .05.

#### 2.3.1 Demographic data

We compared demographic data (biographical data, fitness characteristics) between groups using independent sample *t*‐tests when data were normally distributed and the Mann–Whitney *U* test otherwise.

#### 2.3.2 Pain intensity ratings

To test our H1, we first tested whether there are group differences in the rating of the 38.0°C warm stimulus using a Mann–Whitney *U* test, as data were not normally distributed.

Second, a two‐factorial ANOVA was performed for pain intensity ratings of thermal stimulation during fMRI with the within‐subject factor *Stimulation Intensity* (45, 47, and 48.9°C) and the between subject factor *Group* (athletes, nonathletes). Further, Eta squared η^2^ were calculated to indicate the effect size of any significant effects.

### 2.4 MRI data acquisition and preprocessing

FMRI was performed with a 3T MRI scanner (Siemens Magnetom Prisma fit, Erlangen, Germany) using a 64‐channel standard head coil. The scanner's physiological monitoring system was used to record cardiac and respiratory cycles (peripheral pulse [PPU] and respiratory belt).

Gradient echo EPI was used to acquire T2*‐weighted BOLD data with following parameters: time to echo [TE] = 30 ms, repetition time [TR] = 3,000 ms, flip angle = 90°, matrix = 64 × 64, field of view [FoV] = 192 mm × 192 mm. Each of the 198 volumes comprised 50 axial slices with voxels of 2.4 mm x 2.4 mm × 2.4 mm (x, y, z) resolution which were acquired parallel to the intercommissural plane (AC‐PC plane) covering the entire brain. The total functional scan duration was about 10.25 min. Additionally, a high‐resolution T1‐weighted anatomical scan (3D‐MPRAGE sequence, TE = 3.03 ms, TR = 2,300 ms, 192 slices, resolution = 1 mm x 1 mm x 1 mm [x, y, z]) was acquired to facilitate image co‐registration. For pre‐processing and data analysis, SPM12 (Wellcome Trust Centre for Neuroimaging, London, UK) with MATLAB 2017b (MathWorks, Sherbon, MA) was used. Based on the pre‐processed images, connectivity analyses were performed with the CONN‐fMRI Functional Connectivity toolbox v18a (Whitfield‐Gabrieli & Nieto‐Castanon, 2012).

Preprocessing started with slice timing to correct differences in image acquisition time between slices. Anatomical data of each subject was normalized to the MNI stereotaxic space and co‐registered with the realigned and unwarped functional data using the standard SPM12 algorithms. Finally, spatial smoothing (6 mm full‐width half‐maximum isotropic Gaussian kernel) was applied to the images.

### 2.5 Functional MRI analysis

For first‐level statistical analysis of fMRI data, a general linear model (GLM) approach was used (Friston et al., 1999) with the stimulation intensities “38°C,” “45°C,” “47°C,” “48.9°C” as predictors. Further predictors (of no interest) were “rating” for the rating periods and “rest” for the period subsequent to stimulation (Rest 1, see Figure 2). The time elapsing during “Rest 2” was added to baseline. The PPU and respiratory data of each subject were input to the PhysIO toolbox (Kasper et al., 2017), which was used to calculate time course regressors that modeled variability in physiological noise. A total of three cardiac and four respiratory terms were used along with one interaction term, to create a total of 18 RETROICOR style regressors (Glover, Li, & Ress, 2000). These regressors were included in the first‐level GLM design matrix, along with the six main parameters of head motion, as further predictors of no interest. Additionally, we compared the mean deviation of head movement pre versus post scan, and volume to volume accumulated head movements between groups by conducting two sample *t*‐tests. All six parameters of head movement revealed no significant group difference (all *p* > .1), see Table S1. The expected blood oxygen level‐dependent (BOLD) signal change for each predictor was modeled by a canonical hemodynamic response function. Following the estimation of the GLM using the method of restricted maximum likelihood, statistical parametric maps were generated for the stimulation intensities “38°C,” “45°C,” “47°C,” and “48.9°C” for further use in the second‐level analysis. For second‐level analysis, a GLM with random‐effects approach was used by calculating a Flexible‐Factorial Design as implemented in SPM12 with the within‐subject factor *Stimulation Intensity* (38, 45, 47, and 48.9°C), and the between‐subject factor *Group* (athletes vs. nonathletes). This repeated‐measures GLM also allows to study the interaction of both factors, that is, differences in *Groups* according to *Stimulation Intensity*.

As manipulation check, we calculated the t‐contrast 45°C + 47°C + 49°C > 3*38°C for the whole sample to examine heat versus warm specific fMRI differences.

To test our H2, we first tested whether athletes and nonathletes differ in brain activation during warm stimulation. Therefore, a t‐contrast was conducted, comparing brain activation of nonathletes > athletes during warm stimulation (38°C). Second, we conducted a t‐contrast to compare the brain activation of nonathletes > athletes during painful heat stimulation (45°C + 47°C + 48.9°C).

Additionally, we explored whether the subject's fitness level is associated with the activation strengths of brain regions by calculating spearman correlations (as data were not normally distributed) between the *β* value of the significant brain clusters with LT in Watt per kg body mass (LT [W/kg]) for the whole sample.

To further explore whether there are any fMRI differences among the 45, 47, and 48.9°C stimulation, we exploratively calculated the following t‐contrasts: 48.9°C > 45°C, 48.9°C > 47°C, 47°C > 45°C for the whole sample, respectively.

The summary statistic image was thresholded at uncorrected *p* = .001 with FWE correction at cluster‐level, *p* = .05, based on random field theory (Lieberman & Cunningham, 2009). This resulted in a minimal cluster extent of 28 contiguous voxels.

### 2.6 Functional connectivity analysis

To examine changes in functional connectivity within predefined brain regions that are typically activated by nociceptive stimulation due to *painful heat stimulation* in athletes and nonathletes, ROI‐to‐ROI analyses were performed. Based on previous fMRI‐studies on pain (Apkarian, Bushnell, Treede, & Zubieta, 2005; Simons et al., 2014; Vierck, Whitsel, Favorov, Brown, & Tommerdahl, 2013), 14 regions were included: left thalamus, right thalamus, left postcentral gyrus (PostCG), right (PostCG), left parietal operculum (PO), right PO, ACC, BS, left rostral PFC, right PFC, left anterior Insula, right anterior Insula, left Amygdala, and right Amygdala. Regions were provided by the CONN toolbox (Whitfield‐Gabrieli & Nieto‐Castanon, 2012). All of these regions were also found in the functional contrast as clusters of activation to painful stimulation (45°C + 47°C + 48.9°C) in the comparison between nonathletes > athletes (see Table 2). Additionally to the regions above, we introduced the left and right posterior insula as spheres with a radius of 5 mm. Moreover, we added two spheres with the same radius for two brainstem clusters. Posterior insulae and both brainstem clusters were also activated in the comparison between nonathletes > athletes for painful stimulation (45°C + 47°C + 48.9°C, see above). Prior to the connectivity analysis confounding signals were removed from fMRI data by an anatomic component‐based noise correction (Behzadi, Restom, Liau, & Liu, 2007). Then a 0.008–0.09 Hz temporal band pass filter was applied to the time series. Similar to the first level GLMs described above, six head motion parameters and 18 RETROICOR style regressors from the PhysIO toolbox served as covariates of no interest for each subject to rule out their influences. Corrected time‐series were then used to estimate condition‐based ROI‐to‐ROI connectivity as primary outcome measure (Whitfield‐Gabrieli & Nieto‐Castanon, 2012). Mean signal time courses were extracted within the 14 ROIs separately for each *Stimulation Intensity* and each subject and used to calculate Pearson correlations between ROI pairs of interest. The resulting ROI‐to‐ROI estimates were finally converted using Fisher's Z‐transformation and submitted to a GLM with factors *Group* (athletes vs. nonathletes) and *Stimulation Intensity* (“38°C,” “45°C,” “47°C,” and “48.9°C”), allowing to focus on differences in functional connectivity between both groups in one framework.

**TABLE 2:** Clusters of activation to painful stimulation (45°C + 47°C + 48.9°C) in the comparison between nonathletes > athletes

Brain region	Extent	*t*‐value	MNI coordinates (mm)			Correlation: *β*‐value~ LT (W/kg)	
			x	y	z	rs	*p*‐FDR
**L brain stem**	140	5.417	−6	−28	−34	−.62	<.001
R brain stem		4.270	6	−21	−29		
L brain stem		4.188	−8	−21	−27		
**R middle frontal gyrus**	340	5.409	35	54	14	−.65	<.001
R middle orbital gyrus		4.749	37	56	4		
R middle frontal gyrus		4.518	44	39	24		
**R middle frontal gyrus**	120	5.058	44	25	36	−.54	.002
**L fusiform gyrus**	141	4.915	−30	−2	−34	−.51	.003
L medial temporal gyrus		4.449	−23	6	−34		
**R middle frontal gyrus**	116	4.856	28	44	36	−.52	.002
R ACC		4.536	4	25	33		
R superior frontal gyrus		3.975	18	44	26		
**L postcentral gyrus**	209	4.715	−59	−16	40	−.43	.009
L precentral gyrus		4.300	−61	3	26		
L postcentral gyrus		4.864	−61	−14	33		
R postcentral gyrus		4.838	40	−35	52		
**R thalamus**	139	4.699	1	−28	4	−.60	<.001
L thalamus		4.587	−4	−28	2		
R thalamus		4.121	8	−30	9		
**R superior temporal gyrus**	49	4.599	61	−14	9	−.48	.005
**L IFG/anterior insula)**	115	4.542	−42	13	−3	−.51	.003
L superior temporal Gyrus/posterior insula		3.707	−54	−4	0		
L insula		3.589	−35	10	0		
**R precentral gyrus**	92	4.538	56	−4	33	−.43	.009
R postcentral gyrus		4.212	52	−11	33		
**L cerebellum**	74	4.466	−28	−59	−32	−.55	.002
**R posterior medial frontal**	67	4.450	6	18	52	−.43	.009
R posterior medial frontal		3.871	11	13	60		
R MCC		3.415	13	13	48		
**L superior temporal Gyrus**	54	4.242	−40	−30	2	−.47	.006
L superior temporal Gyrus		3.826	−44	−26	7		
**L cerebellum**	38	4.228	−6	−47	−32	−.55	.002
**L cerebellum**	48	4.051	−13	−78	−27	−.63	<.001
**L putamen**	38	3.923	−16	6	0	−.46	.007
L putamen		3.874	−20	15	0		
**R cerebellum**	37	3.895	4	−69	−36	−.62	<.001

*Note*: Clusters of activation with a voxel threshold of *p* < .001 and a cluster threshold of *p* < .05 (28 contiguous voxels) in MNI coordinates for the maxima of the respective cluster. The corresponding neuroanatomical regions are described as derived from Anatomy Toolbox. Right columns: spearman correlation rs and corresponding *p*‐value (FDR corrected) of *β* value of the significant brain clusters with lactate threshold in watt per kg body mass (LT [W/kg]).

To analyze our H3, we first tested whether athletes and nonathletes differed in network functional connectivity during warm stimulation (38°C). Therefore, a t‐contrast was conducted, comparing functional connectivity of nonathletes > athletes during warm stimulation (38°C). Second, we conducted a t‐contrast to compare the network functional connectivity of nonathletes > athletes during painful stimulation (mean [45°C + 47°C + 48.9°C]). To control for false positive results, we chose network measures for nonparametric network‐level interference (NBS: Network Based Statistics, [Zalesky, Fornito, & Bullmore, 2010]). Networks in the entire ROI‐to‐ROI matrix represent maximal subgraphs of suprathreshold‐connected ROIs (groups of ROIs and suprathreshold effects/connections among them) as a two‐dimensional statistical parametric map. Network size and network mass measures both represent measures of degree/cost of these subgraphs (i.e., number and strength of suprathreshold effects/connections within each graph). Specifically, the network mass is defined as the sum of F‐ or T‐squared statistics over all connections within each cluster. Multiple comparison correction was implemented at the network level (false detection rate [FDR] across multiple networks). For network‐level *p*‐FDR corrected threshold we used *p* < .05 (network‐level *p*‐FDR corrected). Network level inference remains valid when used in combination with an arbitrary initial “height” threshold. For this initial thresholding procedure in the ROI‐to‐ROI matrix, we applied a *p*‐uncorrected threshold of *p* < .05. With this progressive initial threshold, we aimed to find all relevant connections in the network rather than few but powerful connections in order to generalize our findings to the whole connectome of pain processing.

Additionally, we explored whether the subject's fitness level is associated with the functional connectivity by calculating spearman correlations (as data were not normally distributed) between the effect size of the significant network functional connectivity with LT in Watt per kg body mass (LT [W/kg]) for the whole sample.

Lastly, we explored whether the brain activation and functional connectivity are associated by calculating spearman correlations (as data were not normally distributed) between the mean *β* value of all significant brain clusters and the mean effect size of all positive/negative significant network functional connections, respectively.

## 3 RESULTS

### 3.1 Behavioral data

#### 3.1.1 Demographic data

In accordance with our selection criteria shown in Table 1, our high endurance athletes and nonathletes differed in both measured parameters of endurance capacity (higher PWC150, higher LT, all *p* < .001), whereas they did not differ in age or BMI (all *p* > .1). We further compared the PWC150 values of each group with age and gender specific reference percentiles for Cardiorespiratory Fitness from “The German National Health Interview and Examination Survey for Adults (DEGS1)” that was recently published by Finger et al. (2019). In the DEGS1‐study, PWC150 was assessed using exactly the same exercise test protocol like in our study. Based on these data our athletes‐group (PWC150: 3.5 W/kg) lies far above the 97.5th percentile (2.71 W/kg) whereas the nonathletes group (PWC150: 1.6 W/kg) lies between the 25th and 50th percentile (1.50 W/kg and 1.77 W/kg, respectively).

#### 3.1.2 Pain intensity ratings

Hypothesis 1: Lower pain ratings in athletes compared to nonathletes to heat stimuli, but not to warm stimuli.

**FIGURE 3:**
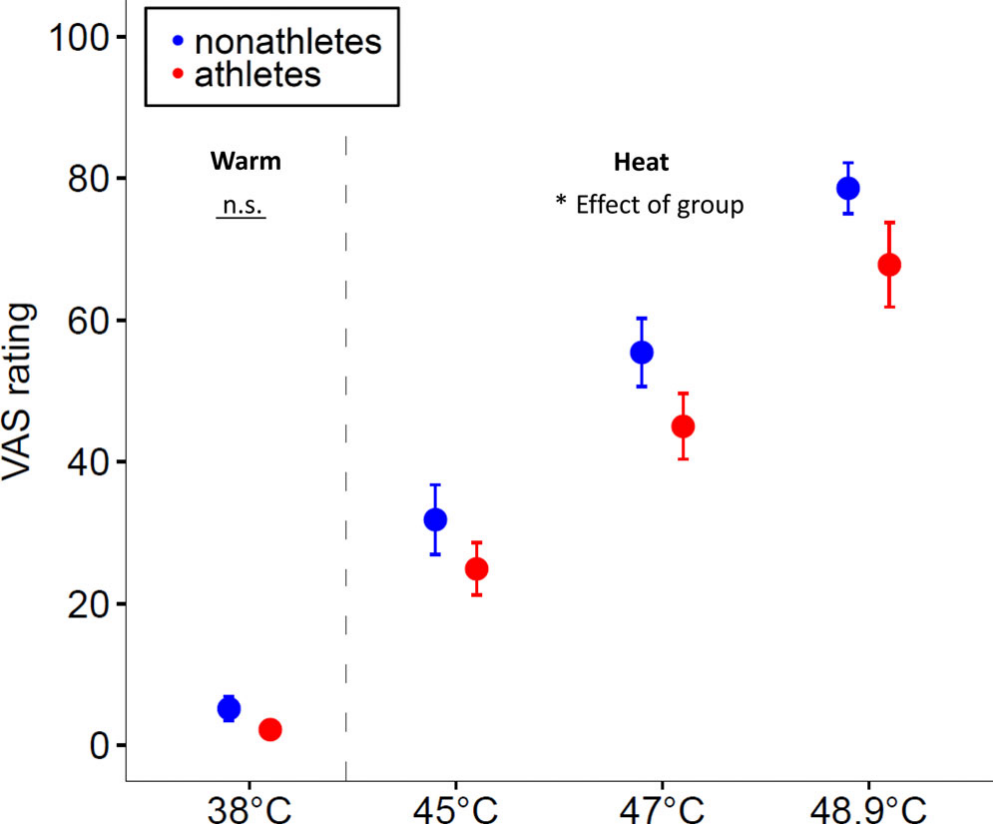
Pain intensity ratings to heat pain and warmth perception in athletes versus nonathletes. Shown are mean and standard errors of pain intensity ratings (0 = no pain, 100 = unbearable pain) to applied stimuli of different intensities. There was no significant group difference in the rating of the 38°C warm stimulus (W = 151, *p* = .518), but a significant group difference in the rating of the heat stimuli. Athletes rated the painful thermal stimulation (45, 47, and 48.9°C) less intense than nonathletes (F(1) = 4.651, *p* = .033, *d* = 0.729). VAS = visual analogue scale. Error bar of 38°C stimulus in the athletes group not visible as the *SE* is nearly 0

The Mann–Whitney *U* test revealed no significant group differences in the rating of the 38°C warm stimulus (W = 151, *p* = .518), suggesting no altered transduction and transmission of thermal signals between groups. See Figure 3.

As expected, the two‐factorial ANOVA of pain intensity ratings revealed a significant main effect of the factor *Stimulation Intensity* (F[2] = 56.604, *p* < .001, *ƞ* ^2^ = 0.618) and of the factor *Group* (*F*(1) = 4.651, *p* = .033, *ƞ* ^2^ = 0.117). Comparisons of mean values showed that athletes rated the painful thermal stimulation less intense (45°C: *M* = 25.7, *SD* = 15.8; 47°C: *M* = 47.1, *SD* = 18.1; 48.9°C: *M* = 71.5, *SD* = 20.7) than nonathletes (45°C: *M* = 31.8, *SD* = 21.4; 47°C: *M* = 55.4, *SD* = 20.9; 48.9°C: *M* = 78.6, *SD* = 15.6). There was no significant interaction of *Stimulation Intensity* and *Group* (*F*(2)= 0.048, *p* = .953). See Figure 3. As the H0 of the Hypothesis 1 could have been rejected, we continued the testing of Hypothesis 2.

### 3.2 MRI data

#### 3.2.1 Functional MRI

The conducted t‐contrast to examine heat (45°C + 47°C + 48.9°C) versus warm (38°C) specific fMRI differences in the whole sample (all participants), as study manipulation check, revealed stronger activation during heat stimulation in several brain regions that are typically activated by nociceptive stimulation bilateral brainstem, bilateral thalamus, bilateral MI, bilateral SII, bilateral anterior and posterior Insula, bilateral ACC, bilateral MCC, and bilateral MFC (see Table S2, and Figure S1).

Hypothesis 2: Reduced activation of brain regions that are typically activated by nociceptive stimulation in athletes compared to nonathletes to painful heat stimuli, but not to warm stimuli.

**FIGURE 4:**
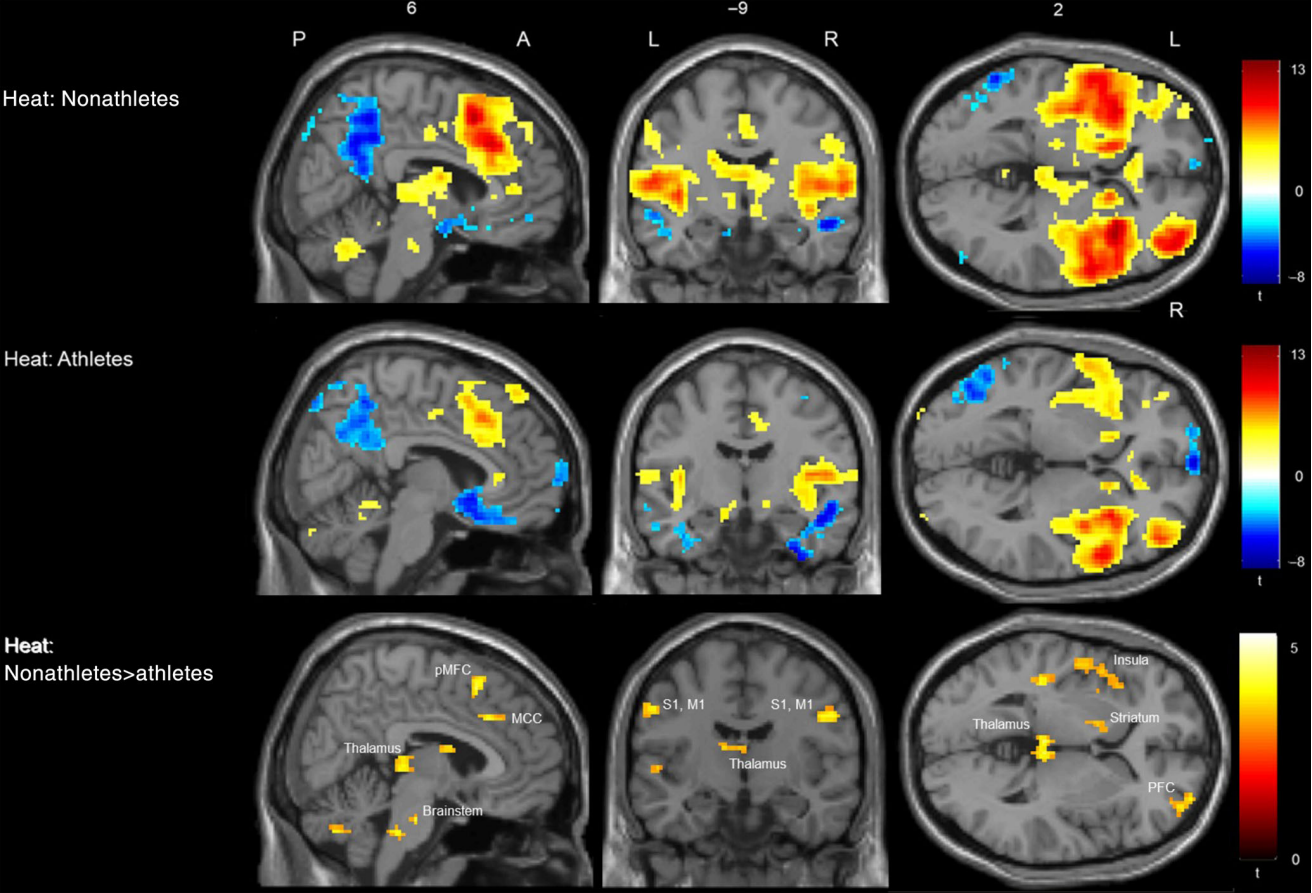
Activation to painful heat stimulation (45°C + 47°C + 48.9°C) in nonathletes, athletes and differences between nonathletes > athletes. Shown are (de)activation clusters to painful heat stimulation (45°C + 47°C + 48.9°C) in the group of nonathletes (top row) and athletes (middle row). The bottom row shows the results of the conducted t‐contrast. Comparing brain activation of nonathletes > athletes during painful heat stimulation. This contrast revealed stronger activation in nonathletes in several brain regions that are typically activated by nociceptive stimulation including bilateral thalamus, bilateral SI and MI, bilateral anterior and posterior Insula, right ACC, bilateral MCC, right PFC, and bilateral brain stem (BS). The summary statistic images were thresholded at uncorrected *p* = .001 with FWE correction at cluster‐level, *p* = .05 based on random field theory. A, anterior site, L, left hemisphere, MCC, midcingulate cortex; MI, primary motor cortex, P, posterior site, PFC, prefrontal cortex; pMFC, posterior middle frontal cortex; R, right hemisphere, SI, primary somatosensory cortex

The conducted t‐contrast, comparing brain activation of nonathletes versus athletes during warm stimulation (38°C), revealed no significant group differences. As expected, the t‐contrast, comparing brain activation of nonathletes > athletes during painful heat stimulation (45°C + 47°C + 48.9°C) revealed only stronger activation in nonathletes in several brain regions that are typically activated by nociceptive stimulation including bilateral thalamus, bilateral SI and MI, bilateral SII, bilateral anterior and posterior Insula, right ACC, bilateral MCC, right PFC, and bilateral BS. There were no brain regions showing significant higher activation in athletes than in nonathletes (See Figure 4 and Table 2). Additionally, we explored whether the activation strengths of these significant brain clusters are associated with the LT. All of the calculated spearman correlations revealed negative associations (all *p* < .05, *p*‐FDR corrected). That means the higher the subject's fitness level the lower the activation strengths of the brain regions during painful stimulation (See Table 2).

To determine whether there are any fMRI differences among the 45, 47, and 48.9°C stimulation, we calculated the following t‐contrasts: 48.9°C > 45°C, 48.9°C > 47°C, and 47°C > 45°C for the whole sample, respectively. Results of these analyses are shown in Tables S3–S5.

As the H0 of Hypothesis 2 could have been rejected, we continued the testing of Hypothesis 3.

#### 3.2.2 Functional connectivity analysis

Hypothesis 3: Different functional connectivity between brain regions that are typically activated by nociceptive stimulation to painful heat stimuli in athletes compared to nonathletes, but not to warm stimuli.

To test our H3, we first tested whether athletes and nonathletes differ in functional connectivity in the predefined network including left thalamus, right thalamus, left PostCG, right PostCG, left PO, right PO, ACC, left BS, right BS, left rPFC, right rPFC, left aInsula, right aInsula, left pInsula, right pInsula, left Amygdala, and right Amygdala during warm stimulation (38°C). The conducted t‐contrast, comparing network functional connectivity of nonathletes > athletes during warm stimulation (38°C), revealed no significant group differences.

**FIGURE 5:**
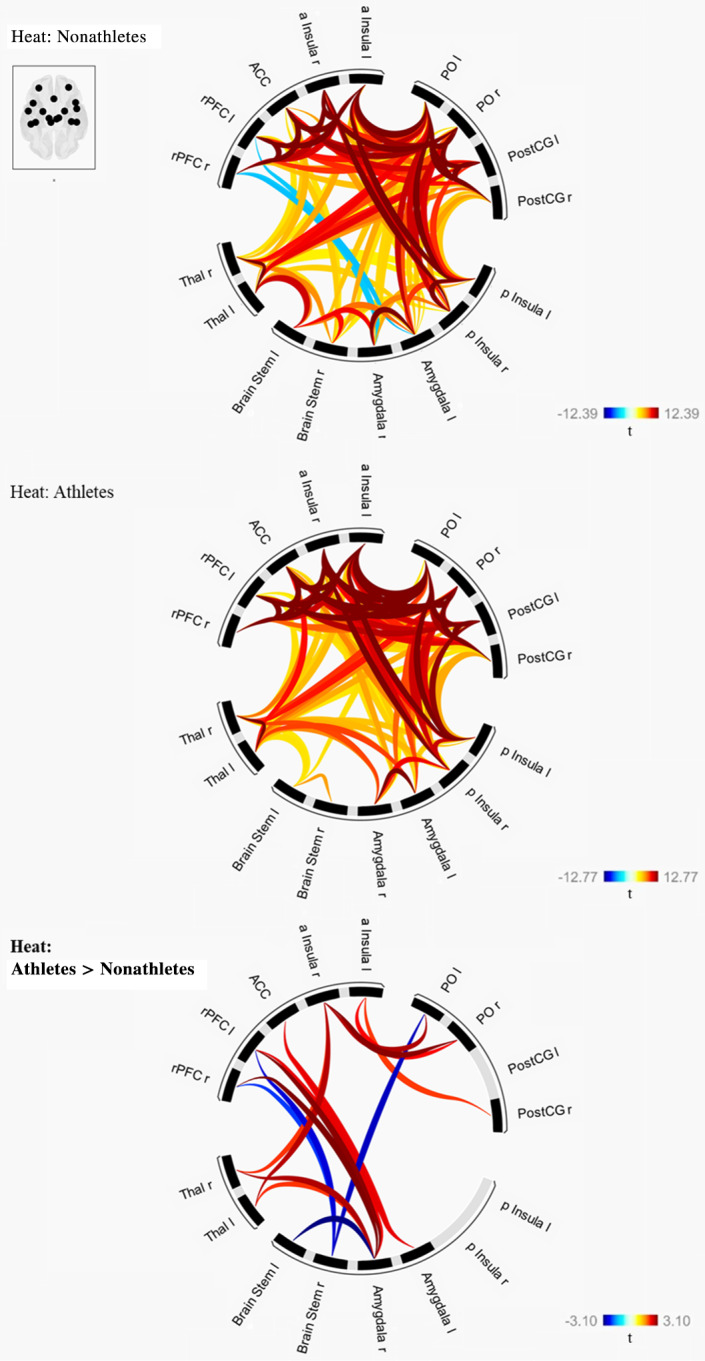
Brain network functional connectivity to painful heat stimulation (mean [45°C + 47°C + 48.9°C]) in nonathletes, athletes and differences between athletes > nonathletes. Shown are network functional connectivity measures to painful heat stimulation (mean [45°C + 47°C + 48.9°C]) in the group of nonathletes (top row) and athletes (middle row). The bottom row shows the results of the conducted t‐contrast, network functional connectivity of athletes > nonathletes during painful heat stimulation. This contrast revealed stronger functional connectivity in athletes between the following ROIs: right Amygdala—right rPFC, right Amygdala—left rPFC, right PO—right aInsula, right Thalamus—right Amygdala, left PO—right aInsula, left Thalamus—right aInsula, left Amygdala—left rPFC, left Amygdala—ACC, right Amygdala—ACC, right PO—left aInsula, right Thalamus—right aInsula, left Thalamus—right Amygdala, right PostCG—left aInsula. In contrast, we only found lower functional connectivity in athletes between left Brain Stem—right Amygdala, right Brain Stem—left PO, right Brain Stem—left rPFC, and right Brain Stem—right rPFC. Initial threshold for all ROI‐to‐ROI connections was set to *p* < .05 (*p*‐uncorrected) and the network‐level *p*‐FDR corrected threshold was set to *p* < .05 (network *p*‐FDR corrected). a, anterior; ACC, anterior cingulate cortex; l, left; PO, parietal operculum; PostCG, postcentral gyrus; r, right hemisphere; ROI, region of interest; rPFC, rostral prefrontal cortex

Second, we conducted a t‐contrast to compare the network functional connectivity of nonathletes > athletes during painful heat stimulation (mean [45°C + 47°C + 48.9°C]). As expected, this analysis revealed significant differences between athletes and nonathletes (Mass statistic = 216.7, *p*‐FDR corrected = .030). Specifically, we found stronger functional connectivity in athletes between the following ROIs: right Amygdala—right rPFC, right Amygdala—left rPFC, right PO—right aInsula, right Thalamus—right Amygdala, left PO—right aInsula, left Thalamus—right aInsula, left Amygdala—left rPFC, left Amygdala—ACC, right Amygdala—ACC, right PO—left aInsula, right Thalamus—right aInsula, left Thalamus—right Amygdala, and right PostCG—left aInsula. In contrast, we only found lower functional connectivity in athletes between left BS—right Amygdala, right BS—left PO, right BS—left rPFC, and right BS—right rPFC (see Table 3 and Figure 5). Notably, the functional connectivities between Amygdala and rPFC result from negative *t*‐values in the nonathletes group but insignificant t‐values in the athletes group.

**TABLE 3:** Brain network functional connectivity to painful heat stimulation (mean [45°C + 47°C + 48.9°C]) in the comparison between athletes > nonathletes

Connection 1	Connection 2	*t*‐value	*p*‐value	Correlation: Effect size~LT (W/kg)	
				rs	*p*‐value
Amygdala r	rPFC r	3.10	.004	.22	.189
Brain stem l	Amygdala r	−2.97	.005	−.31	.064
Amygdala r	rPFC l	2.90	.006	.21	.222
PO r	aInsula r	2.89	.007	.33	**.048**
Thalamus r	Amygdala r	2.71	.010	.45	**.007**
PO l	aInsula r	2.70	.011	.23	.170
Thalamus l	aInsula r	2.67	.011	.29	.092
Brain stem r	PO l	−2.61	.013	−.36	**.031**
Brain stem r	rPFC l	−2.47	.019	−.35	**.034**
Amygdala l	rPFC l	2.38	.023	.25	.138
Amygdala l	ACC	2.36	.024	.29	.089
Amygdala r	ACC	2.31	.027	.14	.421
PO r	aInsula l	2.24	.032	.23	.185
Thalamus r	aInsula r	2.06	.047	.25	.149
Thalamus l	Amygdala r	2.06	.047	.23	.183
Brain stem r	rPFC r	−2.05	.048	−.31	.067
PostCG r	aInsula l	2.04	.049	.35	**.036**

*Note*: Shown are significant differences of the network functional connectivity between athletes > nonathletes during painful heat stimulation (mean [45°C + 47°C + 48.9°C]). To control for false positive results, connection level threshold *p* < .05 (*p*‐uncorrected) and cluster‐threshold *p* < .05 (network *p*‐FDR corrected) has been used. Right columns: spearman correlation rs and corresponding p‐value (uncorrected) of effect size of the significant network functional connectivity with lactate threshold in watt per kg body mass (LT [W/kg]).

Abbreviations: a, anterior site; l, left hemisphere; PO, parietal operculum; PostCG, postcentral gyrus; r, right hemisphere; rPFC, rostral prefrontal cortex.

Additionally, we explored whether the functional connectivity between these ROIs is associated with the LT. The correlation analyses revealed positive associations between the functional connectivity of right PO—right aInsula, right Thalamus—right Amygdala, right Post CG—left aInsula and LT, but negative associations between the functional connectivity of right BS—left PO, right BS—left rPFC and LT (see Table 3).

**FIGURE 6:**
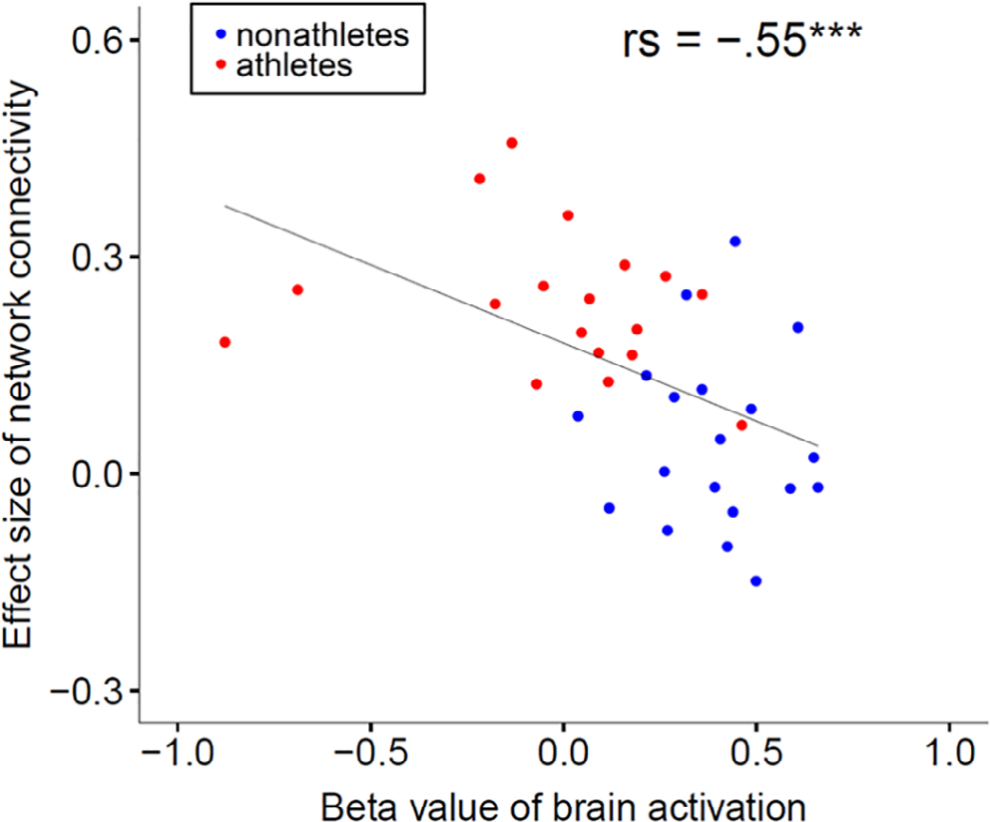
Association of brain activation and network functional connectivity to painful heat stimulation. The correlation between the mean *β* value of all significant brain clusters of the t‐contrast, comparing brain activation of nonathletes > athletes during painful heat stimulation (45°C + 47°C + 48.9°C) and the mean effect size of all functional connectivities between the ROIs that were stronger in athletes compared to nonathletes during painful stimulation revealed a negative association (rs[35] = −.547, *p* < .001). ROI, region of interest

Lastly, we explored whether the brain activation and functional connectivity are associated. We, therefore, calculated for each subject: the mean *β* value of all significant brain clusters of the t‐contrast, comparing brain activation of nonathletes > athletes during painful heat stimulation (45°C + 47°C + 48.9°C), the mean effect size of all functional connections between the ROIs that were stronger in athletes compared to nonathletes during painful stimulation, and the mean effect size of all functional connections between the ROIs that were reduced in athletes compared to nonathletes during painful stimulation. While the correlation between the mean *β* value of brain activation and the mean effect size of all ROIs that were reduced in athletes revealed no significant association (rs[35] = .185, *p* = .271), the correlation between the mean *β* value of brain activation and the mean effect size of all ROIs that were stronger in athletes revealed a negative association (rs[35] = −.547, *p* < .001; see Figure 6).

## 4 DISCUSSION AND CONCLUSIONS

The investigation of the neural mechanisms of pain processing in endurance athletes is important to understand their exceptional abilities to modulate pain. Comparing the pain processing of heat pain stimuli between endurance athletes and nonathletes, our study revealed (a) lower pain intensity ratings in athletes to applied heat stimuli; (b) a reduction of the activation in several brain regions that are typically activated by nociceptive stimulation in athletes to applied painful heat stimuli; (c) stronger functional connectivity between brain regions that are typically activated by nociceptive stimulation to applied painful heat stimuli. However, no such differences were observed for warm stimuli. Lastly, post hoc correlation analyses revealed associations of the subject's fitness level and the brain activation strengths, subject's fitness level and functional connectivity, and brain activation strengths and functional connectivity. Our results suggest that endurance athletes differ in the neural processing of pain elicited by noxious heat.

### 4.1 Heat pain perception in athletes and nonathletes

We used three different heat pain stimulation intensities (45, 47, and 48.9°C) to depict a broad range of pain sensations. Indeed, a two‐factorial ANOVA revealed a significant main effect of the factor *Stimulation Intensity*. Analyzing mean values of pain intensity ratings, we observed a great range of pain intensity rating from *M*_45°C_athletes_ = 25.7 to *M*_48.9°C__non_athletes_ = 78.6. Furthermore, we detected a significant main effect of factor *Group*. Comparisons of mean values showed that athletes rated the painful thermal stimulation generally less intense than nonathletes, which is in accordance with our Hypothesis H1 and several studies reporting higher pain (tolerance) thresholds and lower pain intensity ratings in endurance athletes (Freund et al., 2013; Geva & Defrin, 2013; Tesarz et al., 2012). In the present study, we applied heat pain as noxious stimulus, although endurance athletes mainly have to deal with muscle pain. Elevated pain thresholds and lower pain intensity ratings have already been reported for cold pressure pain (Freund, Weber, et al., 2013; Pettersen, Aslaksen, & Pettersen, 2020; Tesarz et al., 2012), ischemic pain (Tesarz et al., 2012), electrically induced pain (Guieu, Blin, Pouget, & Serratrice, 1992), heat pain (Geva & Defrin, 2013; Pettersen et al., 2020), pressure pain (Flood, Waddington, Thompson, & Cathcart, 2017; Geisler et al., 2020), and chemically induced pain (Johnson, Stewart, Humphries, & Chamove, 2012). Thus, our results and previous reports suggest that the type of painful stimulation has no influence on the described hypoalgesia in athletes. To see whether athletes and nonathletes differ in their ratings of nonpainful stimuli, we additionally performed a Mann–Whitney *U* test of the 38°C warm stimulus. This test revealed no significant group differences, indicating an unaltered transduction or transmission of thermal signals between groups. Lastly, there was no significant interaction of *Stimulation Intensity* × *Group*. In a meta‐analysis, Tesarz et al. (2012) could show that athletes possess higher pain tolerance thresholds than nonathletes, whereas differences of pain thresholds were less consistent across studies. Our results indicate no influence of stimulation intensity on group differences during thermal stimulation. However, our results do not contradict the results of the meta‐analysis by Tesarz et al. (2012) as we did not directly measure pain (tolerance) thresholds.

### 4.2 Brain activation to heat pain stimulation in athletes and nonathletes

Second, we compared the brain activation of nonathletes > athletes during painful heat stimulation (45°C + 47°C + 48.9°C). In line with our Hypothesis H2, this analysis revealed reduced activation in several brain regions that are typically activated by nociceptive stimulation including thalamus, SI and SII, Insula, ACC, MCC, DLPFC, and BS in endurance athletes. Furthermore, exploratively conducted correlation analyses revealed negative associations between the activation strengths of these brain regions and the LT. Thus, higher subject's fitness levels are associated with lower activation strengths of brain regions during painful stimulation. To evaluate whether these differences are specific to the painful range, we also compared the brain activation of nonathletes versus athletes during warm stimulation (38°C). No significant group differences were observed.

Importantly, although meta‐analyses of brain imaging studies revealed that noxious stimuli elicit activity most commonly within SI, SII, Insula, ACC, MCC, PFC, thalamus and BS (Apkarian et al., 2005; Peyron, Laurent, & Garcia‐Larrea, 2000), and that the activation strengths of most of these regions correlate with pain intensity rating (Coghill, McHaffie, & Yen, 2003), it has to be kept in mind that these structures are not exclusively activated by nociceptive stimulation, but also by, that is, nonnociceptive salient sensory input (Iannetti & Mouraux, 2010). A study by Johnson et al. (2012) shows that marathon runners possess higher pain specific self‐efficacy than nonathletes explaining their higher pain tolerance thresholds. Furthermore, Geva and Defrin (2013) revealed that triathletes had lower fear of pain than nonathletes, which was inversely correlated with CPM effect. These results point to the idea that endurance athletes normally evaluate arising pain (i.e., pain due to aching or acute overload) as no threat, knowing they can deal with pain successfully. This learned association (pain—no threat) might lead to a reduced salience of new painful stimuli compared to nonathletes, who have less experiences with pain and therewith no association between pain and threat (and also compared to chronic pain patients, who show increased pain catastrophizing [Edwards, Bingham 3rd, Bathon, & Haythornthwaite, 2006]). Additionally, habituation to nonthreatening pain experiences can contribute to a generally reduced salience of painful stimuli. Lastly, it is conceivable that endurance athletes do not only have more experiences with pain than nonathletes, but also a greater range of pain experiences. If the strongest pain ever experienced is higher in athletes than in nonathletes, then light or moderate painful stimuli are probably evaluated as less salient. Therefore, we suggest that the reduced salience of new painful stimulation might explain to some extend the reduced activation of the brain regions that are typically activated by nociceptive stimulation in athletes. In addition, the obtained results can be associated with movement‐induced hypoalgesia. Movement‐induced hypoalgesia is an immediate effect of sensory attenuation of nociceptive inputs related to voluntary movement with multiple neural mechanisms (Lu, Yao, Thompson, & Hu, 2021). During the fMRI scan, participants were asked to lay motionless. Therefore, we can exclude any short‐term effects of movement‐induced hypoalgesia. However, it is possible that long‐term effects of movement‐induced hypoalgesia might contribute to differences in brain activation between athletes and nonathletes. Another mechanism that might have contributed to the obtained brain data of the present study is CPM. It has been shown that endurance athletes have a greater CPM effect compared to nonathletes (Flood et al., 2016; Geisler et al., 2020; Geva et al., 2017; Geva & Defrin, 2013). Therefore, it is conceivable that endurance athletes have a more efficient top‐down modulation of painful stimuli that leads to a reduced signal input of new presented noxious stimuli and therewith to a reduced brain activity. However, it seems unlikely that CPM has had a direct influence on the outcomes of the present study, as we did not apply a second painful stimulus as conditioned stimulus during painful stimulation. Further studies that additionally assessed the association between the individual exposures to pain during their exercise, the duration of the career, CPM effects, and pain ratings or brain activation during painful stimulation might give more insights to these exciting issues.

### 4.3 Functional connectivities in athletes and nonathletes

To assess whether the functional connectivity between brain regions that are typically activated by nociceptive stimulation is another factor that contributes to different pain perception in athletes, we lastly analyzed the functional connectivity between these brain regions during painful heat stimulation in athletes and nonathletes. In line with our H3, the performed ROI‐to‐ROI analyses revealed stronger functional connectivity between most of the brain regions that are typically activated by nociceptive stimulation during painful heat stimulation in athletes, while there were no group differences during the warm stimulation. Contrary to the stronger functional connectivity between most of the analyzed brain regions, there were reduced functional connectivities between the BS and amygdala, BS and rPFC, and BS and PO in endurance athletes.

Interestingly, Bär et al. (2016) showed after a 6 weeks high‐intensity intervention in young healthy men that functional connectivity between the right ventral hippocampus and the dorsal vagal complex decreased, while an increase was observed for other cortical structures. Since the authors observed a highly significant negative correlation between increased vagal function after exercise and this decreased functional connectivity, a relation to adaptation processes during exercise was suggested. Furthermore, it has been shown previously that the amygdala has a direct connection to the brainstem that gets activated during fear and anxiety to vegetatively prepare a flight or fight response (e.g., increase of HR and respiratory rate, [LeDoux, 2000; Tovote et al., 2016]). If endurance athletes have a reduced association of acute painful stimuli with fear, then the activation of the amygdala‐brainstem pathway to prepare a flight or fight response is less necessary. Future studies are needed to investigate the interaction between BS nuclei and neocortical regions in more detail.

Endurance athletes need to and possibly learned to predict, control, and modulate arising pain over the years of extensive training to become successful (Geva & Defrin, 2013; Johnson et al., 2012). Compared to nonathletes, they are highly motivated to train within a power range that induces pain as these training sessions increase physical fitness very effectively (Gillen & Gibala, 2013). It has been described that motivation has a strong influence on the perception of pain (Wiech & Tracey, 2013). Based on the concept of neural networks and correlation learning (Hebb, 1949), we suggest that frequent highly motivated pain experiences might lead to stronger bidirectional connectivity between brain regions that are typically activated by nociceptive stimulation in endurance athletes. This is also in line with the idea of pain as a driver of reinforcement learning (Seymour, 2019). Specifically, the reinforcement learning theory states that pain signal guides prospective behavior to minimize harm through learning. With regard to endurance athletes, it is conceivable that they use unconsciously a very efficient endogenous pain inhibition during their training sessions and as this behavior is used repeatedly, it leads to adaptive changes in the network that process pain.

Alternatively, our fMRI results can be interpreted in line with the predictive coding theory (Buchel, Geuter, Sprenger, & Eippert, 2014). This theory states that the brain uses sensory inputs including pain to continuously optimize the model or prediction of the world. The main goal is to minimize the free energy or prediction error, which is the mismatch of descending predictions (expectation) and incoming sensory data (observation) (Buchel et al., 2014; Friston, 2009). Importantly, this theory is based on a Bayesian framework describing prior predictions and observed sensory signals as probability density functions. This means that the precision (inverse variance of the probability function) of the prediction and observed sensory signal mainly influences the amount of the prediction error. If endurance athletes have more experiences in pain than nonathletes, they also might have a more precise probability function of the sensory input pain. Provided that, the probability function of the pain prediction (expectation regarding painful stimulation) is the same between athletes and nonathletes, this should result in a reduced prediction error. In other words, endurance athletes might have developed a better model of pain due to their greater pain experiences. Together, the results of our fMRI data suggest that endurance athletes process painful stimuli by activating a stronger connected brain network with simultaneously reduced activation strength of the brain regions that are typically activated by nociceptive stimulation. The negative association between the brain activation strength and functional connectivity strengthens this conclusion. Both phenomena may explain the reduced pain perception in endurance athletes described in literature (Tesarz et al., 2012) and replicated in our study. Furthermore, the results of the post hoc correlation analyses between the LT and brain activation strength, and the LT and functional connectivity support the idea that the subject's fitness level is an important variable that can influence functional brain alterations. In summary, the obtained results from this study strengthen the validity of the reinforcement learning theory as well as the predictive coding theory.

### 4.4 Limitations and further directions

Our study has several limitations. First, we did not blind the experimenter, as there were partly too obvious signs that allowed the experimenter to guess whether a subject is an athlete (strong muscles, very low heart frequency during rest, etc.) or not. However, as the instructions were the same for participants from both groups and as all participants were naïve to the hypotheses of the study, we think that the results of our study are reliable. In particular, we found differences between athletes and nonathletes not only in subjective pain rating, but in the neural processing of pain, an objective measure. Second, we included two unspecific BS clusters in our network analysis, although the BS is quite a big structure and there are specific regions that are certainly more important for the processing and modulation of pain than others. We, therefore, recommend future studies to use a higher spatial resolution of fMRI data to explore the specific role of different nuclei of the BS in more detail. Third, we reported that athletes showed lower brain activation and stronger functional connectivity between brain regions during painful heat stimuli, but not during warm stimuli. However, the nonsignificant results of the warm stimuli might be influenced by the lower number of trials and the resulting reduced signal to noise ratio. Fourth, it is possible that changes in the brain activity could be partly result of changes in peripheral or spinal processing of the thermal stimuli (that we did not assess) before these signals reach the brain. However, we could show that athletes and nonathletes also differ in the functional connectivity of brain regions processing thermal stimuli. Although this result does not provide any conclusive evidence, it strengthens the assumption that athletes differ in the neural processing of pain elicited by noxious heat. Fifth, the data of our study does not allow to differentiate between nociception, pain, and unpleasantness, as we did not measure these parameters in separated conditions. Future studies are highly recommended to focus on differences between athletes and nonathletes in nociceptive processing versus pain perception, as it has been shown that they are processed via two dissociable brain systems (Woo, Roy, Buhle, & Wager, 2015). Sixth, it is possible that the presented results might be influenced by the type of endurance athletes. Indeed, different sport types are associated with different characteristics of pain perception and modulation, as well as of thoughts toward pain. Whereas endurance‐based sports are associated with improved pain inhibition, strength‐based sports are more associated with reduced pain sensitivity (Assa, Geva, Zarkh, & Defrin, 2019). We examined runners and triathletes and thus endurance athletes of the most common types of endurance activities (running, cycling, and swimming). Although triathlon is in some terms different in context of the movement patterns to the individual sports of swim, bike and run, physiological adaptations as well as aerobic capacity seem to be quite equal. Therefore, a potential influence of the subtype of endurance activity was not taken into account.

Lastly, our study was designed as a cross‐sectional study. Therefore, we cannot conclude whether endurance athletes develop differences in the neural processing of pain due to extensive training or whether only persons with already extraordinary abilities to modulate pain become endurance athletes. Longitudinal studies are highly recommended to answer this key question.

In summary, the results of the present study indicate that endurance athletes do not only differ in subjective measures of pain perception like pain ratings but also in the activation strength of the brain regions that are typically activated by nociceptive stimulation and the functional connectivity between these brain regions during painful stimulation. Furthermore, it seems that pain experiences could not only have negative influences on pain perception, as it has been shown according to surgery, traumas, or chronic intensive care treatment, but that pain experiences in a positive context like endurance sport can even increase pain (tolerance) thresholds and make the processing of nociceptive stimuli in the brain more efficient. Together, these results are not only important from a scientific point of view as they expand the current theory of pain processing, but moreover they might contain valuable information to get deeper insights in chronic pain states and might open a window for potential strategies to modulate pain in clinical pain conditions. Additional studies are needed to investigate the effective connectivity and structural connections between the brain regions of interest, the role of neurotransmitters, and psychological variables on pain perception in athletes.

## CONFLICT OF INTERESTS

The authors declare no competing financial interests.

## AUTHOR CONTRIBUTIONS

Maria Geisler, Alexander Ritter, Marco Herbsleb, Karl‐Jürgen Bär, and Thomas Weiss made a significant contribution to the conception, design of experimental study, the analysis, and interpretation of data, and participated in drafting the manuscript or reviewing, and/or revising it for intellectual content.

## Supporting information


**Appendix** **S1**: Supporting Information

## Data Availability

The data that support the findings of this study are available from the corresponding author upon reasonable request.
