# Supplementary material for: Neural mechanisms of pain processing differ between endurance athletes and nonathletes: A functional connectivity magnetic resonance imaging study
Source: Hum Brain Mapp. 2021 Sep 15;42(18):5927–42. doi: 10.1002/hbm.25659 (PMC8596969; doi:10.1002/hbm.25659)
Supplement: Supplementary file 1 — Appendix S1: Supporting Information [file HBM-42-5927-s001.docx]

**Table S1: Descriptive Statistics of head movement during fMRI scan**

|  | **athletes** | | | | | | **non-athletes** | | | | | | |
| --- | --- | --- | --- | --- | --- | --- | --- | --- | --- | --- | --- | --- | --- |
|  | translation (mm) | | | rotation (°) | | | translation (mm) | | | rotation (°) | | | |
|  | x | y | z | pitch | roll | yaw | x | y | z | pitch | roll | yaw |  |
| Mean: pre vs. post scan | 0.13 | 0.20 | 0.39 | 0.009 | 0.004 | 0.003 | 0.19 | 0.26 | 0.54 | 0.013 | 0.007 | 0.005 |  |
| sd | 0.18 | 0.43 | 0.35 | 0.010 | 0.005 | 0.002 | 0.19 | 0.34 | 0.44 | 0.015 | 0.007 | 0.005 |  |
| Mean: accum. volumes | 9.6 | 25.8 | 30.1 | 0.021 | 0.005 | 0.002 | 11.2 | 18.8 | 47.7 | 0.033 | 0.009 | 0.006 |  |
| sd | 20.4 | 79.1 | 35.6 | 0.034 | 0.008 | 0.004 | 17.7 | 53.9 | 62.6 | 0.060 | 0.018 | 0.013 |  |

To test whether athletes and non-athletes differ in the degree of head movement, we analyzed all 6 parameters of head movements (three translation parameters (x, y and z-axes) and three rotation parameters (pitch, roll, and yaw)), respectively. First, we compared the mean deviation of head movement pre vs. post scan between groups by conducting two sample t-tests. All 6 parameters of head movement revealed no significant group difference (all p > 0.1). To get values that represent the total amount of movement during the fMRI scan, (as the first analysis neglects short head movement peaks), we secondly analyzed the volume to volume accumulated head movements. To bring all values on a positive scale, we squared the values of each parameter of each subject and each volume. We then accumulated the squared values of all volumes for each subject, respectively. Again, the conducted two sample t-tests revealed no significant group differences of the 6 accumulated movement values between groups (all p > 0.1).

**Table S2.**

**Clusters of activation to heat (45°C, 47°C, 48.9°C) > warm (38°C) stimulation.**

|  |  |  | MNI Coordinates (mm) | | |
| --- | --- | --- | --- | --- | --- |
| Brain Region | Extent | t-value | x | y | z |
|  |  |  |  |  |  |
| R Insula | 19626 | 9.765 | 35 | 6 | 9 |
| R Insula |  | 9.511 | 35 | -16 | 19 |
| L Insula |  | 8.881 | -35 | 1 | 12 |
| R Insula |  | 4.039 | 49 | 13 | 2 |
| L Rolandic Operculum |  | 3.423 | 49 | -16 | 21 |
| R Putamen |  | 3.198 | 32 | -6 | -3 |
| L Cerebellum | 68 | 5.482 | -25 | -38 | -41 |
| L Precentral Gyrus | 52 | 4.534 | -54 | 8 | 50 |
| R Middle Frontal Gyurs | 188 | 4.531 | 47 | 3 | 57 |
| R IFG | 188 | 3.618 | 54 | 13 | 38 |
| L Precentral Gyrus | 81 | 4.455 | -40 | -6 | 62 |
| R Superior Temporal Gyurs | 87 | -4.393 | 59 | -62 | 26 |
| R Middle Temporal Gyrus | 38 | -4.041 | 54 | -2 | -20 |

Clusters of activation with a voxel threshold of p<0.001 and a cluster threshold of p<0.05 (28 contiguous voxels) in MNI coordinates for the maxima of the respective cluster. The corresponding neuroanatomical regions are described as derived from Anatomy Toolbox.


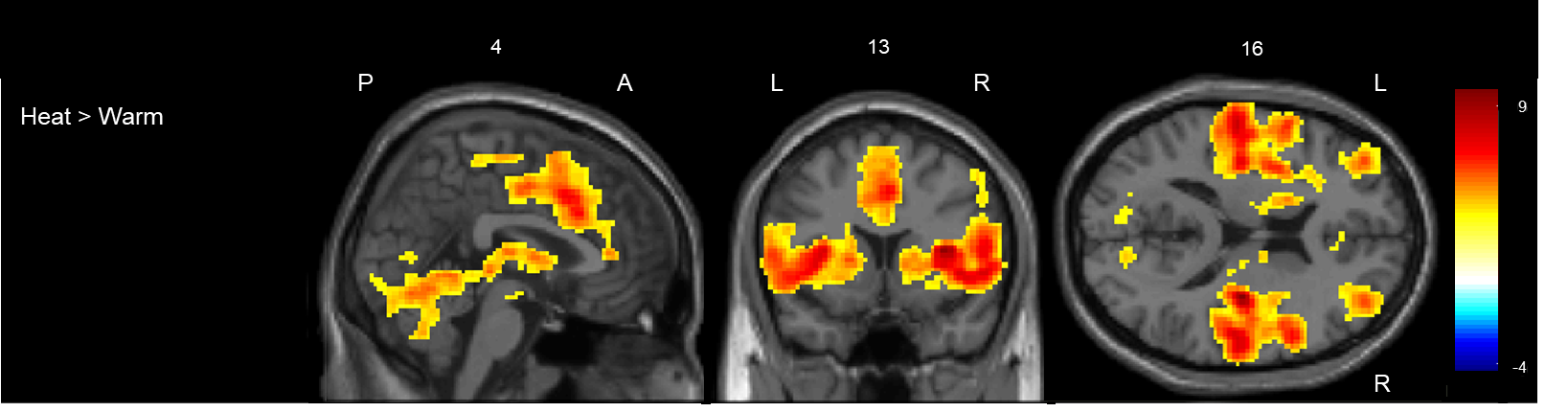


**Fig. S1: Activation to painful heat (45°C + 47°C + 49°C) > warm (38°C) stimulation.**

Shown are activation clusters to painful heat (45°C + 47°C + 48.9°C) > warm (38°C) stimulation in the group of non-athletes and athletes. This contrast revealed stronger activation during heat stimulation in several brain regions of the neurosignature of physical pain including bilateral brainstem, bilateral thalamus, bilateral MI, bilateral SII, bilateral anterior and posterior Insula, bilateral ACC, bilateral MCC, bilateral MFC. The summary statistic images were thresholded at uncorrected *p* = 0.001 with FWE correction at cluster-level, *p* = 0.05. based on random field theory. R = right hemisphere, L = left hemisphere, P = posterior site, A = anterior site.

**Table S3.**

**Clusters of activation to heat stimulation in the comparison between 48.9°C > 45°C.**

|  |  |  | MNI Coordinates (mm) | | |
| --- | --- | --- | --- | --- | --- |
| Brain Region | Extent | t-value | x | y | z |
|  |  |  |  |  |  |
| L Insula | 140 | 5.706 | -32 | 3 | 12 |
| L anterior Insula |  | 4.320 | -40 | 8 | -3 |
| L Putamen |  | 3.970 | -30 | -6 | 12 |
| L Cerebelum | 408 | 5.457 | -13 | -57 | -17 |
| R Insula | 288 | 4.076 | 49 | 1 | 7 |
| R MCC | 624 | 5.300 | 6 | -11 | 43 |
| Cerebellar Vermis | 74 | 4.990 | -1 | -64 | -34 |
| L Rolandic Operculum | 175 | 4.799 | -59 | 1 | 12 |
| L Temporal Pole |  | 3.802 | -54 | 6 | 0 |
| L Insula | 194 | 4.790 | -40 | -18 | 19 |
| L Rolandic Operculum |  | 4.152 | -49 | -16 | 19 |
| L Supramarginal Gyrus |  | 3.832 | -61 | -26 | 21 |
| R Middle Frontal Gyurs | 68 | 4.464 | 44 | -4 | 62 |
| R Precentral Gyrus |  | 3.400 | 54 | -4 | 52 |
| R Superior Temporal Gyrus | 128 | 4.397 | 47 | -33 | 21 |
| R Rolandic Operculum |  | 3.650 | 56 | -18 | 19 |
| R Inferior Temporal Gyrus | 34 | 4.257 | 52 | -74 | -3 |
| L Cerebelum | 408 | 3.524 | -6 | -64 | -17 |
| L Inferior Parietal Lobule | 138 | -3.902 | -37 | -66 | 57 |
| R Middle Temporal Gyrus | 51 | -3.770 | 71 | -35 | -3 |
| R Superior Temporal Gyrus |  | -3.518 | 61 | -26 | 0 |

Clusters of activation with a voxel threshold of p<0.001 and a cluster threshold of p<0.05 (28 contiguous voxels) in MNI coordinates for the maxima of the respective cluster. The corresponding neuroanatomical regions are described as derived from Anatomy Toolbox.

**Table S4.**

**Clusters of activation to heat stimulation in the comparison between 47°C > 45°C.**

|  |  |  | MNI Coordinates (mm) | | |
| --- | --- | --- | --- | --- | --- |
| Brain Region | Extent | t-value | x | y | z |
|  |  |  |  |  |  |
| L Insula | 41 | 4.500 | -32 | -18 | 19 |
| R Postcentral Gyrus | 71 | 4.120 | 44 | -23 | 43 |
| R Inferior Temporal Gyrus | 61 | 3.944 | 52 | -74 | -3 |
| R MCC | 32 | 3.827 | 8 | 8 | 43 |
| R Putamen | 34 | 3.809 | 32 | -18 | 12 |
| R Insula |  | 3.671 | 45 | -16 | 21 |
| R Superior Temporal Gyrus | 47 | 3.687 | 49 | -28 | 21 |
| R Rolandic Operculum |  | 3.552 | 52 | -18 | 28 |

Clusters of activation with a voxel threshold of p<0.001 and a cluster threshold of p<0.05 (28 contiguous voxels) in MNI coordinates for the maxima of the respective cluster. The corresponding neuroanatomical regions are described as derived from Anatomy Toolbox.

**Table S5.**

**Clusters of activation to heat stimulation in the comparison between 49°C > 47°C.**

|  |  |  | MNI Coordinates (mm) | | |
| --- | --- | --- | --- | --- | --- |
| Brain Region | Extent | t-value | x | y | z |
|  |  |  |  |  |  |
| Cerebellar Vermis | 221 | 4.492 | -1 | -64 | -34 |
| L Cerebelum |  | 4.313 | -13 | -62 | -20 |
| R Angular Gyrus | 78 | -3.882 | 35 | -66 | 50 |

Clusters of activation with a voxel threshold of p<0.001 and a cluster threshold of p<0.05 (28 contiguous voxels) in MNI coordinates for the maxima of the respective cluster. The corresponding neuroanatomical regions are described as derived from Anatomy Toolbox.
